# Questioning the Extreme Neurovirulence of Monkey B Virus* (Macacine alphaherpesvirus 1)*

**DOI:** 10.1155/2018/5248420

**Published:** 2018-02-13

**Authors:** R. Eberle, L. Jones-Engel

**Affiliations:** ^1^Department of Veterinary Pathobiology, Center for Veterinary Health Sciences, Oklahoma State University, Stillwater, OK 74078, USA; ^2^Department of Anthropology and Center for Studies in Ecology and Demography, University of Washington, Seattle, WA 98195, USA

## Abstract

Monkey B virus (*Macacine alphaherpesvirus* 1; BV) occurs naturally in macaques of the genus* Macaca, *which includes rhesus and long-tailed (cynomolgus) monkeys that are widely used in biomedical research. BV is closely related to the human herpes simplex viruses (HSV), and BV infections in its natural macaque host are quite similar to HSV infections in humans. Zoonotic BV is extremely rare, having been diagnosed in only a handful of North American facilities with the last documented case occurring in 1998. However, BV is notorious for its neurovirulence since zoonotic infections are serious, usually involving the central nervous system, and are frequently fatal. Little is known about factors underlying the extreme neurovirulence of BV in humans. Here we review what is actually known about the molecular biology of BV and viral factors affecting its neurovirulence. Based on what is known about related herpesviruses, areas for future research that may elucidate mechanisms underlying the neurovirulence of this intriguing virus are also reviewed.

## 1. Introduction

Herpesviruses are ubiquitous viruses, found in a wide variety of species including mammals, birds, and reptiles. Three subfamilies of the Herpesviridae family (Alpha-, Beta- and Gammaherpesvirinae) are found in the order Primates. Of these, alphaherpesviruses typically infect and remain within the peripheral sensory nervous system for the life of their host as part of their natural life cycle. The close and prolonged association of these viruses with their host over its entire lifetime with only rare impairment of nervous system function implies an exquisite degree of host-virus coadaptation. On occasion stability of this commensal symbiotic host-virus relationship can be altered, resulting in severe or even fatal disease often involving the central nervous system (CNS). In cases where an alphaherpesvirus infects a host of another species, the result can be, but is not always, catastrophic. The most notorious example of this is monkey B virus (*Macacine alphaherpesvirus* 1; BV), an alphaherpesvirus enzootic in macaques of the genus* Macaca*. Though exceptionally rare, zoonotic BV infection following exposure to macaques has a mortality rate of ~80%. Here we review what is known about this relatively neglected virus with regard to its infamous neurovirulence.

## 2. BV in Its Natural Host

Monkey B virus (BV) occurs naturally in all 17 species of macaque monkeys that comprise the genus* Macaca. *Macaques are ecologically adaptable monkeys and are the most numerous and widely distributed nonhuman primate on the planet. The majority of macaque species are distributed throughout Asia and their ubiquity has led to three of these species (*M. mulatta*,* M. fascicularis, *and* M. nemestrina*) being used as biomedical research models for nearly a century [1–4]. Although known by several names over the years since its initial isolation in 1932 (*Herpesvirus simiae,* monkey B virus, Herpes B, and* Cercopithecine herpesvirus *1), BV is currently designated as* Macacine alphaherpesvirus* 1 by the International Committee on Taxonomy of Viruses.

From a biological standpoint, in macaques BV is very much like herpes simplex virus (HSV) in humans. Both HSV and BV are normally transmitted horizontally via direct contact and exchange of bodily secretions [3, 5–7]. The prevalence of BV in macaques is related to age, increasing progressively from infant to adult [8–17]. There are very few studies on the prevalence or transmission of BV in free-ranging populations. In captive macaque colonies, very young monkeys (<2 yr of age) usually acquire BV as an oral infection, while in socially and reproductively mature macaques primary BV infections are usually genital. The prevalence of BV in adults of both wild populations and captive breeding colonies typically ranges from 70% to nearly 100%. Only rarely does BV cause lethal infections in healthy macaques, just as HSV rarely causes encephalitis or other serious disease in humans [18].

As with HSV in humans, most primary BV infections occur in mucosal epithelium and do not usually produce overt clinical signs, although lesions are sometimes visible on close inspection [27]. As the virus replicates in epithelial cells, sufficient levels of infectious virus accumulate allowing the virus to invade unmyelinated sensory nerve endings present in the epidermis. Once in sensory neurons, the virus establishes a latent infection in the sensory ganglion, where the viral genome is retained in the nuclei of neurons without entering the lytic viral replicative cycle that occurs in epithelial cells [6, 28–30]. Viral replication in epithelial tissue is eventually controlled and the virus is eliminated by the host adaptive immune response. The only indication that a monkey continues to harbor BV is the presence of circulating antiviral IgG. It has been shown in captive macaques that, in rare instances, usually in infants or the very young, primary infections may not end with the establishment of latency, but rather progress as generalized infections that spread throughout the body and are frequently fatal [31–34].

As is typical of other alphaherpesviruses, BV can periodically reactivate from the latent state in response to various stressful stimuli, resulting in shedding of infectious virus. While lesions may be apparent, most recurrences do not produce clinically apparent lesions; rather, infectious virus is shed asymptomatically. Like HSV in humans, the frequency of BV shedding appears to be fairly low (2-3%) [35–37]. Stress related to social/housing challenges, transportation, immunosuppression, and seasonal breeding have all been linked to reactivation of latent BV [6, 7, 38, 39].

In many ways, BV is the macaque equivalent of human HSV: BV and its macaque hosts have coevolved, resulting in an exquisitely fine-tuned interaction with one another that results in perpetuation of the virus within the host's nervous system with minimal adverse effects on the host but also with occasional shedding of infectious virus that can be transmitted to a naïve host, thereby ensuring perpetuation of the virus. The notorious neurovirulence of BV is therefore not evident in its natural macaque hosts; the neurovirulence of BV only becomes apparent when BV infects other species, particularly humans.

## 3. Zoonotic BV Infection

In 1932 a young physician, William Brebner, performing poliovirus research with rhesus macaques was bitten on the finger [40–42]. He developed herpetic-like lesions on the finger, and the infection eventually progressed to involve the CNS. The patient died several weeks later from an acute ascending myeloencephalitis. A herpesvirus was isolated from several tissues at autopsy. Although initially identified as HSV, the virus was subsequently shown to be distinct from HSV and was designated as “the B virus” [40, 42].

While the exact number is not available, less than 60 additional cases of pathogenic zoonotic BV infection have occurred sporadically over the last 85 years, all resulting from exposure to laboratory or captive macaques or macaque tissues [2, 4, 5, 43–50]. While zoonotic BV infections are exceedingly rare, the fatality rate is 70–80% and many survivors are left with deteriorative neurologic sequelae. The majority of exposures have been associated with bites or scratches from captive, laboratory-housed macaques. However, additional modes of exposure have been implicated including mucosal membrane contact with macaque urine and/or feces, needlestick injury, and contamination of cuts with material from primary macaque cells in the laboratory [43, 44, 48]. With the sole exception of one spousal person-to-person infection [47], all zoonotic BV infections have involved primate veterinarians, animal care personnel, or laboratory researchers in North America working with macaques or macaque biologics. BV is the single most serious occupational zoonotic concern for persons working with or around macaques.

While the clinical course of zoonotic BV infections can vary, initial symptoms usually develop within 1–3 weeks of an exposure incident [51]. The nature of initial clinical symptoms also varies but usually includes nonspecific flu-like symptoms, vesicular herpetic lesions at the site of exposure, and symptoms indicative of involvement of the peripheral and/or central nervous systems. The infection progressively spreads along sensory nerves into the spinal cord and ascends into the brainstem. Typically, the destruction of nervous tissue as the virus spreads within the CNS results in encephalomyelitis and respiratory failure in terminal stages of the infection. Once the CNS is involved, the final outcome is almost invariably death.

Though most persons known to be infected with BV die, some do survive and some survivors can periodically shed virus after recovery [46, 52–54]. Several cases of zoonotic BV have also occurred in persons who have a history of working with macaques but without any known BV exposure immediately prior to the appearance of clinical signs. Both of these observations suggest that BV latency not only occurs in humans, but that reactivation of latent BV can be associated with clinical disease [47, 54, 55]. Since primary BV and HSV infections in the natural host species are usually asymptomatic, the potential exists for asymptomatic zoonotic BV infections to occur as well. However, there has only been one study that tested persons working with captive macaques for serological evidence of BV infection [56]. None were detected, suggesting that asymptomatic infections are likely uncommon, if they do occur.

A puzzling aspect of zoonotic BV infections is the notable lack of any fatal or even clinically evident BV infections in Asia where BV-positive macaques and humans have copious close interactions, and exposure to macaque bodily fluids through bites, scratches, and mucosal splashes is common [11, 19, 57–59]. The human-macaque interface is diverse and deep in Asia, in part because of cultural and religious beliefs that provide a context for tolerance and a measure of protection for these ubiquitous monkeys. Macaques, particularly the abundant rhesus, long-tailed and pigtail monkeys, are found at the thousands of temples and shrines located throughout a broad geographic swath extending east from Afghanistan to Japan and south through the Indonesian archipelago. Millions of people who live and work at these sites, as well as those who worship, have frequent contact with macaques. Additionally, many of these sacred sites are also international tourist destinations, drawing hundreds of thousands of visitors each year who come to appreciate the culture and to feed and interact with the monkeys. Human exposures are routine, with macaques aggressively pursuing food handouts while climbing on visitors. Bites and scratches commonly occur, especially to international tourists who lack experience with monkeys, when humans either fail to relinquish food or behave in a manner the monkeys deem threatening. In a retrospective study of French tourists seeking medical treatment for an animal bite received in Southeast Asia, most reported that the injuring animal was a monkey [60]. Studies have shown that between 6 and 40% of visitors to a monkey temple will be bitten, and thus it is not surprising that zoonotic transmission of a primate retrovirus (simian foamy virus) has been documented following exposure to macaques in Asia [20, 61–63].

In addition to the hundreds of monkey temples across Asia, tens of thousands of macaques are free-ranging in urban areas such as Singapore, Hong Kong, Delhi, and the famous wild monkey parks of Japan. Macaques are also commonly found as pets, and a centuries-old tradition of keeping and training performing monkeys continues in China and Japan. Finally, it should be noted that Indonesia, Thailand, Malaysia, Singapore, Vietnam, Cambodia, Laos, Philippines, China, and Japan all have active biomedical research programs and/or primate breeding research centers which collectively employ thousands of workers and involve tens of thousands of macaques each year. Many of these breeding facilities operate under conditions of extreme animal overcrowding with husbandry and handling protocols that are substandard (Jones-Engel, pers. observ.). Despite frequent contact between humans and free-ranging, temple, pet, or urban macaques in Asia, fatal cases of BV have only occurred in the US and Canada following contact with captive macaques. This geographic restriction of zoonotic BV infections has long been and remains a puzzle.

One immediate question is whether or not BV is even zoonotically transmitted in Asia. There is no question that individuals in communities living near macaques or who work in SE Asian monkey forests have a history of extensive macaque contact and injury [19]. If such persons had experienced BV infection over their years in close contact with wild macaques, their antiviral serum antibodies should differ in their virus-specificity from that of persons never exposed to BV. When an HSV infected person is infected with an antigenically related virus, an anamnestic response will occur to antigens shared by the two viruses, while a* de novo* response will occur to antigens specific to the second virus. Thus, detection of BV-specific antibodies is a difficult problem, as the BV-specific antibody response develops more slowly and may be overshadowed by the immediate and strong response to cross-reactive antigens. Consequently, persons infected with HSV who had experienced an asymptomatic BV infection would be expected to have higher levels of antibodies directed against antigens shared by all primate alphaherpesviruses than would be present in sera of persons only infected with HSV.

**Figure 1:**
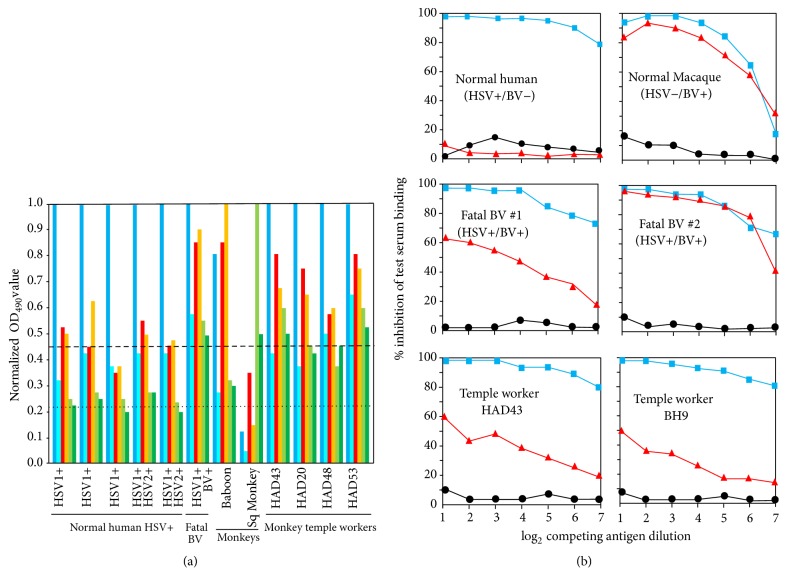
Evidence of possible asymptomatic BV infections in Asia. (a) Sera from individuals working at monkey forests in SE Asia [19, 20] were tested by ELISA as described [21, 22] against HSV1 (dark blue), HSV2 (light blue), BV (red), HVP2 (orange), squirrel monkey herpesvirus (light green), and spider monkey herpesvirus (dark green) antigens. HSV1 OD values were normalized to 1.0 to assess relative levels of reactivity with cross-reactive antigens. Average levels of cross-reactivity with BV and HVP2 in normal HSV-positive control sera (individuals with no known contact with monkeys) are indicated by the dashed line, and average levels of cross-reactivity with the two S. American monkey viruses are indicated by the dotted line. The four sera from monkey forest workers and serum from a fatal case of zoonotic BV infection have higher levels of cross-reactivity with all simian virus antigens than do control sera. (b) Competition ELISAs were performed as described [23, 24] to determine if sera from monkey forest workers with high levels of cross-reactivity were consistent with having been infected with BV. Soluble antigens (extracts of cells infected with HSV1 (blue), BV (red), or uninfected cells (black)) were used to compete the binding of serum to HSV1 antigen coated onto the ELISA plates. Binding of control HSV1-positive serum was inhibited only by soluble HSV1 antigen, not by BV or control antigens. Binding of BV-positive macaque serum (HSV-negative) to the solid phase HSV1 antigen was equally competed by soluble HSV1 and BV antigens. Binding of sera from two patients that died of zoonotic BV infection (both HSV1-positive) were competed by both HSV1 and BV soluble antigens, although competition by BV antigen was less than by HSV1 antigen. Binding competition for sera from two monkey forest workers (both HSV1-positive) was similar to that of zoonotic BV patient sera.

Limited testing compared the relative reactivity of sera from persons working in monkey forests with that of persons having no known contact with monkeys (negative controls) and patients that died of zoonotic BV infection (positive controls) (Figure 1). When sera were tested by ELISA against HSV and multiple simian virus antigens, it was evident that a few monkey forest workers had higher levels of reactivity with simian virus antigens than was evident in most other monkey forest workers or negative control sera (Figure 1(a)). Such elevated antibody levels directed against cross-reactive alphaherpesvirus antigens suggests that these individuals have experienced an infection with a virus antigenically related to but different from HSV. Further analysis by sensitive competition ELISA [23, 24] confirmed that the reactivity of these sera was consistent with that of having been infected with BV (Figure 1(b)). Given the absence of any history consistent with typical BV infection (i.e., infections with neurological involvement), it is possible that these persons experienced asymptomatic BV infections. However, if asymptomatic BV infections do occur in Asia, then the lack of apparent asymptomatic BV infections in the US presents a different enigma.

When assessing the neurovirulence of BV, it is important to recognize that, within Asia where hundreds of thousands of macaques come into daily contact with millions of people, there is no conclusive evidence of zoonotic infections, neurological or otherwise. Genetic differences in human subjects (Asian versus non-Asian background) would seem an unlikely explanation for the lack of fatal BV infections in Asia since many non-Asian tourists visiting monkey forests in Asia and non-Asian military troops serving in Asia have experienced bites and scratches from macaques without any resulting zoonotic BV infections [11, 19, 61, 64]. Similarly, inaccurate diagnosis of zoonotic BV infections in rural areas with limited healthcare seems unlikely explanation as tens of thousands of macaques are free-ranging in large metropolitan areas in Asia where access to healthcare, diagnostics, and case follow-up are readily available (e.g., Singapore, Hong Kong, and Kyoto). It is even less likely that clinicians in a tourist's home country would fail to diagnose BV when presented with a history of a macaque bite and neurological symptoms. Despite being exposed to populations of macaques known to be BV positive, no cases of zoonotic BV have been reported among international tourists [65].

Perhaps a more likely explanation lies in the monkeys, that is, captive versus free-ranging. Do captive macaques shed BV more frequently as a result of some husbandry practices? When they are shedding do they shed more virus? Do free-ranging monkeys preferentially shed virus (or more virus) genitally rather than orally, while captive monkeys shed more orally? All these are questions that have been examined only superficially or not at all. It is particularly intriguing that amongst the dozens of primate breeding facilities that have operated in Asia for decades, some of which contain up to 10,000 macaques, there have been no reported cases of zoonotic BV. The macaques housed in these facilities are exported and are a source of animals used in biomedical research in North America. Other possibilities such as recombinant BV in captive monkeys that arose through past practices of cohousing of different species during capture and shipping have also been raised. The lack of BV isolates from free-ranging macaques for comparison to BV isolates from captive macaques and isolates recovered from zoonotic cases will be necessary to address these and many other aspects of BV neurovirulence.

## 4. Model Systems for Zoonotic BV Infection

Rabbits have historically been used as an animal model for BV infections. Until recently, all testing of antivirals for anti-BV activity was conducted using rabbits [66–70]. Rabbits are however not an ideal model system given their size, the relative paucity of immunological reagents, and the difficulty of housing and handling infected animals under stringent biohazard conditions. Infant mice were found to be susceptible to BV and using this model, the spread of BV in an axonal-transsynaptic manner was demonstrated [71, 72]. However, this model has a number of inherent drawbacks (immature immune system, size, and dependable numbers/availability) and has not been used further. Infection of young adult mice by intramuscular (i.m.) injection (similar to a bite wound) found substantial variation in the neurovirulence of various strains of BV isolated from rhesus monkeys, but this model system was not highly reproducible [73]. However, inoculation of young adult Balb/c mice by skin scarification of the flank was found to produce disease very similar to that seen in humans and to be very reproducible [74]. This mouse model has recently been used for testing efficacy of antiviral drugs and molecular studies on BV [75–77]. Lesions in the brainstem of BV infected mice are characterized by perivascular cuffing with mononuclear cells, discrete foci of neuronal necrosis, gliosis, and discrete areas of destruction of white matter within reticular tracts, accompanied by large foamy macrophages (gitter cells) similar to those present in spinal cord lesions. Viral antigen is also present within neurons and glial cells, and the severity of inflammation is related to the amount of viral antigen present [73]. The inflammatory response to infection has been linked to the lethality of HSV encephalitis in mice [78, 79], and an aggressive inflammatory response in neural tissue may well contribute to the lethality of BV infections in both mice and humans.

In this mouse model, all BV isolates from rhesus macaques and an isolate from a long-tailed macaque* (M. fascicularis)* were found to have similar LD_50_ values of approximately 10^4^ PFU [77]. In contrast, isolates from pigtail* (M. nemestrina)* and lion-tailed macaques* (M. silenus)* were not lethal (at 10^6^ PFU) despite producing clinical signs of neurological involvement. This is interesting when one considers that most if not all cases of zoonotic BV have been associated with exposure to rhesus or long-tailed macaques rather than pigtail macaques (lion-tailed macaques are endangered and not used in biomedical research).

As mentioned above, the neurovirulence of BV actually relates to its neurovirulence in nonnatural host species. In this regard, it is interesting that no zoonotic infections due to the two viruses most closely related to BV, HVP2 of baboons* (Papio *spp.) and SA8 of vervets* (Cercopithecus aethiops)*, have been reported (with one probable exception [50]). Both baboons and vervets have long been used in biomedical research and exposure incidents have certainly occurred. It thus appears that when SA8 or HVP2 are transmitted to humans they both undergo abortive infections. It is thus interesting that most HVP2 isolates have been shown to be just as neurovirulent as BV in mice [74, 80] and to be the cause of a lethal neurological infection in a black-and-white colobus monkey* (Colobus guereza)* [81]. Given the lower biosafety rating of HVP2 (BSL2 versus BSL4 for BV), HVP2 is an attractive model for BV cross-species infections [74, 76].

## 5. Molecular Aspects of BV

From the time of its original isolation in 1932, extensive antigenic cross-reactivity between BV and HSV has been noted, indicating that these viruses are closely related [42, 82–85]. Subsequent studies revealed that alphaherpesviruses of baboons (HVP2), vervets (SA8), and chimpanzees (ChHV) and to a lesser extent the viruses of squirrel monkeys (HVS1) and spider monkeys (HVA1) are all closely related [23, 83, 85–90]. Phylogenetic analyses of the alphaherpesviruses based on gene sequences have defined three major clades of primate alphaherpesviruses consisting of the hominid viruses (HSV1, HSV2, and ChHV), cercopithecine (African and Asian) monkey viruses (BV, HVP2, and SA8), and the platyrrhine (S. American) monkey viruses (HVS1 and HVA1) [91–93]. Based on the close relatedness of BV and HSV, the relative lack of research on the simian viruses, and the biohazard concerns in working with infectious BV, comparatively little experimental molecular work with BV has been published. Thus, most of what is “known” about BV structure, protein functions, and viral replication is actually extrapolationed from what is known for HSV. However, as more work is done with BV and related simian viruses, significant differences between these viruses and HSV become more apparent.

BV has the typical virion structure of alphaherpesviruses, the genome being enclosed within an icosahedral capsid that is embedded in an amorphous protein tegument and surrounded by a lipid membrane envelope [51, 94]. Like HSV, the lytic replication cycle of BV is rapid with extracellular progeny virus appearing ~6–8 hrs after infection (PI) [95]. And as for HSV, synthesis of BV proteins appears to follow the immediate early/early/late gene expression paradigm.

**Figure 2:**
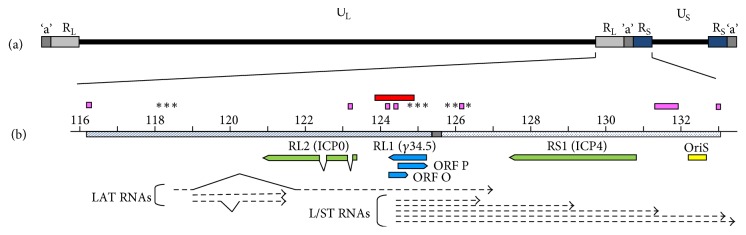
Genomic organization of BV. The BV genome is comprised of two unique regions (U_L_ and U_S_) in which open reading frames homologous to the UL1–UL56 and US1–US12 genes of HSV are located, respectively (a). The U_L_ and U_S_ regions are each flanked by repeat regions (R_L_ an R_S_, resp.) with the ‘a' repeat present at the ends of the genome and between the internal copies of R_L_ and R_S_. The R_L_ and R_S_ regions are enlarged in (b) to indicate the position of the following features discussed in the text: predicted ORFs (green), HSV ORFs not found in BV (blue), the origin of DNA replication in R_S_ (yellow), miRNAs (asterisks), islands of reiterated sequences (magenta), and the region deleted in the E90ΔRL1 mutant (red).

In 1971, an isolate of BV from a rhesus macaque was adapted to replicate in primary rabbit cells, and this strain (E2490) now serves as the “standard” or “laboratory” BV strain [69, 96]. The genome sequence of this strain has been determined [97–99]. Recently, genome sequences of a number of additional BV strains from various macaque species have also been determined [77, 100]. BV genomes range in size from 154,958 to 157,447 bp, the differences being largely due to variation in the number of iterations of repeated sequence units in specific areas of the genome. The BV genome has a very high G + C content (~75%) and its genetic arrangement is orthologous to that of HSV (Figure 2). Based on PCR/sequencing of a small region of the BV genome, different “genotypes” of BV were identified that correlated with the macaque species the virus was isolated from [24, 101, 102]. Comparison of complete genome sequences of BV isolates from different macaque species confirmed the division of BV into host species-based genotypes [77]. While sequence identity among BV isolates from rhesus macaques or between isolates from pigtail macaques is >99%, sequence identity among different BV genotypes is only ~89–95%. Comparing the genome sequences of all BV isolates, most coding sequences, miRNAs, and small RNAs are highly conserved [77, 100, 103, 104]. The most prominent differences are located in areas of the long and short repeat regions (R_L_ and R_S_, resp.) of the genome that do not encode proteins, miRNAs, or small RNAs. Within these areas, there are islands of reiterated sequences. While the primary sequence of these reiterated repeat units and the number of iterations are not conserved among all BV isolates, the positions of these repeat islands are conserved suggesting they likely serve some unknown function.

The genome of BV (as well as HVP2 and SA8) is very similar to that of HSV2 and ChHV in its genetic organization, with homologs of almost every HSV gene being present in the same order and orientation in BV [77, 92, 99, 105, 106]. There are some minor differences like the grouping of some genes into different cotranscriptional units [107]. However, there is one major difference, that being the lack of a detectable homolog of the RL1 (*γ*34.5) and ORF P genes in the simian viruses [92, 99, 105, 106]. This was somewhat unexpected as both of these genes have been shown to be involved in neurovirulence of HSV (see below). It should be noted however that none of the alphaherpesviruses of nonprimate animals have homologs of the RL1 or ORF P genes either.

Based on DNA sequence data, variation in predicted amino acid (AA) sequence identity of homologous BV and HSV2 proteins averages 62.5% [99]. In contrast, average AA sequence identity values are approximately 95% among BV strains, 87% between BV and HVP2, and 83% between BV and SA8 [100, 105, 106]. This level of AA sequence homology is consistent both with previous studies that detected antigenic cross-reactivity of almost all BV proteins with homologous proteins of HSV [83, 87, 108–111] and with the extensive antigenic cross-reactivity observed between BV and HSV in ELISA, western blot, and neutralization assays [84–86, 112–115].

The highly conserved nature of most HSV, ChHV, and simian virus proteins argues for the homologous proteins of each virus having similar functions. Certainly this is true for structural capsid proteins (which as a functional group are the most conserved proteins) and viral enzymes. However, a number of BV proteins have regions of dissimilarity relative to other simian viruses and even among different genotypes of BV. Given the interaction of viral immediate early (IE) proteins with host cell proteins to facilitate expression of the viral DNA and initiate the lytic replication cycle, it might be expected that the IE proteins would be highly conserved. However, these regulatory IE proteins are actually some of the least conserved, both among BV genotypes and between BV and other primate viruses. By virtue of their expression on the surface of virions and infected cells, glycoproteins and other membrane associated proteins are another group of proteins that likely interact with elements of host cells. While some are strongly conserved (>90% AA sequence identity), others are very poorly conserved (<62% AA identity). Consistent with this, a number of glycoproteins have some degree of virus-specific antigenicity. The gB (UL27) and gD (US6) glycoproteins are major immunogens of BV, and while both are structurally conserved and have many cross-reactive epitopes, each also has some degree of antigenic BV-specificity [70, 87, 108, 109, 116, 117]. Both the gG (US4) and gC (UL44) glycoproteins are much less conserved and are largely BV-specific antigens with respect to HSV, but still exhibit some antigenic cross-reactivity with the homologous glycoproteins of HVP2 and SA8 [25, 109, 110, 116, 118–120]. Regardless of the degree of conservation/divergence of BV proteins from those of other related viruses, with one exception there are no studies where the involvement of specific BV proteins in neurovirulence has been examined. The one exception is the UL41 gene which encodes the virion host shutoff (VHS) protein. Deletion of this ORF does not cause a significant reduction in the LD_50_ of BV in mice [75].

While BV encodes homologs of all the various HSV proteins mentioned above, little is known about these BV proteins regarding functional equivalency to their HSV counterparts. BV glycoproteins gC (UL44) and gD (US6) as well as VHS (UL41) have been shown to have similar structural and functional properties to their HSV homologs [75, 117, 120–122]. While AA homology suggests that BV proteins function much as their HSV homologs do, the functional equivalency of all other BV proteins has not been directly examined. As one example, the BV ICP0 (RL2) homolog has the characteristic RING finger domain and many other structural motifs of the HSV ICP0 (phosphorylation sites, USP7/ND10 localization region, nuclear localization signal, and multiple SIM-like sequences [123]). While this certainly indicates that the BV ICP0 protein is structurally very similar to the HSV ICP0 protein, the actual functional equivalency of BV ICP0 has yet to be demonstrated. Furthermore, while these various structural motifs are evident, even minor differences in their primary AA sequence could have an effect on their differential function in epithelial versus neuronal cells or macaque versus human-macaque cells.

Although not examined in BV, the UL39 gene is interesting with regard to host-specific neurovirulence. Despite the lack of any clinical differences in its natural baboon host, phylogenetic analyses and testing in mice place HVP2 isolates into two distinct subtypes [26, 80, 124]. One subtype (HVP2ap) produces no clinical signs of infection in mice following infection with as much as 10^6^ PFU but does induce an adaptive immune response. In contrast, isolates of the second subtype (HVP2nv) are extremely neurovirulent in mice, having an LD_50_ of ~10^4^ PFU (like BV). This dichotomous mouse-specific neurovirulence phenotype of HVP2 isolates allowed mapping of the neurovirulence locus. Recombinant HVP2ap/nv viruses were constructed and tested for neurovirulence in mice. Correlation of the neurovirulence phenotype with genome sequence analyses (defining which parts of the genome were derived from HVP2ap versus HVP2nv) identified a limited region of 3-4 genes that was associated with the neurovirulence phenotype. Based on this, the UL39 gene was the most likely candidate for the “neurovirulence gene,” and this was ultimately confirmed by construction and testing of UL39 ORF-specific recombinant viruses [125]. Thus, replacing the UL39ap coding sequence with the UL39nv coding sequence makes an HVP2ap virus neurovirulent, and vice versa.

The UL39 gene encodes the large subunit of the ribonucleotide reductase protein (R1). UL39 has been associated with neurovirulence in HSV (the ability to replicate in nondividing nerve cells) and is one of several genes deleted from prototype HSV gene delivery vectors [126]. While the C-terminal ~75% of the R1 protein is highly conserved and has homology to mammalian ribonucleotide reductase proteins, the N-terminal regions of the HSV, HVP2, and BV R1 proteins are unrelated to other known proteins and (in HSV) are not essential for ribonucleotide reductase enzymatic activity [127, 128]. It is this N-terminal region that is distinct in the two HVP2 subtypes, implying that the N-terminal region of the UL39 protein in some way determines the neurovirulence of HVP2 in mice. Interestingly, this region of BV UL39 exhibits similarly extensive sequence variation between BV isolates from rhesus/long-tailed macaques (that are neurovirulent in mice) and pigtail/lion-tailed macaque isolates (that are not lethal in mice). It remains to be seen if UL39 underlies neurovirulence of BV in mice as it does in HVP2.

The HSV R1 protein is involved in evasion of cell death by preventing necroptosis in human but not mouse cells [129–131]. In mouse cells, the HSV R1 protein interacts with receptor interacting kinase 1 (RIP1) and RIP3 via their RIP homotypic interaction motifs (RHIMs) which is also present in the N-terminal region of HSV R1, and this interaction ultimately leads to formation of necrosomes and death of the infected cell. In human cells, R1 disrupts the interaction between RIP1 and RIP3, also in a RHIM-dependent manner, thereby preventing necroptosis. Both the BV and HVP2 R1 proteins contain this RHIM motif, and the sequence in this small area is highly conserved. There is however one subtype-specific AA substitution in the RHIM of HVP2ap/nv R1, but the AA residue present in nonneurovirulent HVP2ap isolates is the same as that present in all BV isolates that are lethal in mice, suggesting that this AA may not be responsible for the HVP2 neurovirulence phenotype.

## 6. Does BV Have a Functional Homolog of the HSV RL1 (***γ***34.5) Neurovirulence Gene?

In HSV both the RL1 (*γ*34.5) gene encoding the ICP34.5 (RL1) protein and the ORF P gene (on the opposite strand and overlapping the RL1 gene) have been shown to be primary determinants of neurovirulence in mice [132–137]. Given the neurovirulent reputation of BV, the finding that BV lacks homologs of both the RL1 and ORF P genes was not expected. The HSV RL1 coding sequence starts approximately 200 bp from the internal copy of the ‘a' repeat (Figure 2). However, no ATG initiation codon is apparent within 500 bp of the ‘a' repeat in any BV strain (or in HVP2 or SA8). Similarly, no potential termination codon is present in any of the simian viruses near the initiation point of the L/ST RNAs where the HSV RL1 termination codon is located. Furthermore, no predicted AA sequence homology exists in the “RL1 region” in any of the simian viruses. Thus, multiple investigators have been unable to identify a simian virus homolog of the RL1 or ORF P genes [77, 99, 100, 105, 106]. Despite the lack of a discernable simian homolog of the HSV RL1 gene (or an overlapping ORF P gene on the opposite strand), the RL1 region between the RL2 start codon and the ‘a' repeat of the simian virus genomes (1844–2146 bp) is about the same size as in HSV and ChHV (2048–2154 bp). This suggests that despite the lack of discernable homologs of the RL1 and ORF P genes, this region of the simian virus genomes serves some function. While there are no apparent ORFs in this region of BV, there are two islands of reiterated sequences, and RNA structural analysis of this region (which includes the 5′ part of the L/ST RNAs) indicates a plethora of potential stem-loop secondary structures. The sequence in this region is highly conserved among 15 strains of BV from rhesus macaques but not between BV isolates from different macaque species. Whether or not this region encodes functions similar to those of HSV is not known.

Comparing the RL1 region sequences of simian virus genomes, there are some conserved features. As in HSV2 and ChHV, the three simian viruses all have three miRNAs, a potential TATA sequence, and both ICP4 and SP1 binding site motifs that are likely control elements for transcription of L/ST RNAs [138]. There is also a potential ORF P start codon located approximately 760–815 bp 5′ of these putative L/ST transcriptional control elements in all three simian viruses (in HSV the ORF P start codon is 3′ of the L/ST control elements). The translated sequence from this start codon in BV is conserved only for the initial four codons (M-A-A-R/E), after which no discernable sequence homology exists. Whether this possibly represents an actual start codon to a spliced gene or is simply the result of sequence similarity due to the extremely high G + C content of this region (producing a strong bias towards high-GC codons) is unknown. It may also be that this potentially conserved AA sequence is completely irrelevant, conserved DNA sequence instead representing part of the conserved transcriptional start site of the L/ST RNAs.

**Figure 3:**
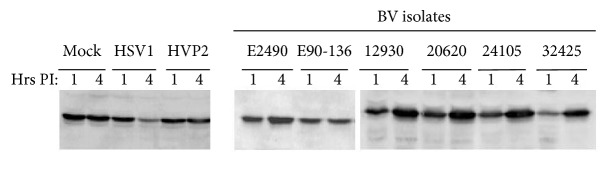
Lack of eIF2*α* dephosphorylation by BV. Cells were harvested at 1 or 4 hrs PI and western blots were performed with antibody directed against phosphorylated eIF2*α* (Cell Signaling Technologies; Danvers, MA) as described [25]. No change in the level of phosphorylated eIF2*α* was evident in mock infected cells and the amount of phosphorylated eIF2*α* decreased from 1 to 4 hrs PI in HSV1 infected cells. In contrast, phosphorylated eIF2*α* levels increased in BV infected cells.

HSV mutants lacking the RL1 or ORF P genes are attenuated in a number of mouse model systems, and they fail to spread within the nervous system even when inoculated directly into the brain [132–134, 137]. The ICP34.5 protein plays a crucial role in allowing HSV to evade the host innate immune response by blocking MHC II expression on the surface of infected cells. When infected, cells initiate an antiviral interferon response that primarily affects neighboring uninfected cells making them more resistant to infection, and ICP34.5 deletion mutants are very sensitive to this host antiviral IFN-*α*/*β* response [139–141]. Another host cell response to infection is activation of double-stranded RNA-dependent protein kinase (PKR). PKR acts to phosphorylate translation initiation factor eIF-2*α* resulting in cessation of protein synthesis in the cell. The HSV ICP34.5 protein dephosphorylates eIF-2*α*, thus counteracting eIF-2*α* phosphorylation-induced autophagy [142–146]. The C-terminal region of ICP34.5 has homology with GADD34 protein which facilitates its interaction with the MyD116 and PCNA cell proteins to form a DNA-binding complex [139, 146, 147]. ICP34.5 thus appears to be of central importance to the HSV replicative cycle in many ways. Given the importance of ICP34.5 functions in HSV, the close relatedness of BV and HSV, and the similar size of the RL1 region of the genomes, it is reasonable to hypothesize that this region of the genome may serve some similar function(s) in all these viruses. However, unlike HSV none of the simian viruses display the ICP34.5 function of eIF-2*α* dephosphorylation following infection (Figure 3). Quite the opposite, accumulation of phosphorylated eIF-2*α* is readily evident in BV infected cells. How BV continues to replicate while eIF-2*α* is phosphorylated is not known.

**Figure 4:**
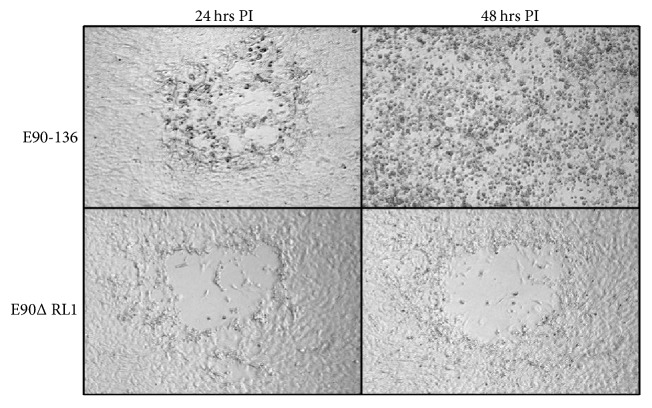
Deletion of the RL1 region affects BV spread in primary mouse cells. Primary murine skin fibroblast cells were prepared, cultured, and infected as described [26]. At low MOI (0.3 PFU/cell) wild-type BV (E90-136) forms plaques by 24 hr PI and rapidly spreads over the next 24 hrs, while the E90ΔRL1 deletion mutant fails to spread after 24 hrs PI.

Using the deletion/replacement approach described for other genes [75, 125], a BV mutant (E90ΔRL1) lacking 1162 bp in the RL1 region but retaining 5′ transcriptional control elements of the RL2 (ICP0) gene and 2 of 3 miRNAs located adjacent to the ‘a' repeat was constructed. This mutant grows as well as the parental wild-type virus in Vero cells, indicating that this region of the genome is not essential for BV replication* in vitro*. When proteins synthesized at various times PI were examined (1-2, 4–6, 10–12, and 4–24 hrs PI), no differences were detectable between the parental wild-type virus and the E90ΔRL1 mutant. In primary mouse skin fibroblasts infected at a low multiplicity of infection (MOI; 0.3 PFU/cell), wild-type BV forms small plaques by 24 hrs  PI and subsequently spreads to adjacent cells involving the entire cell monolayer by 48 hrs PI (Figure 4). In contrast, while the E90ΔRL1 mutant also forms small plaques by 24 hrs PI, these fail to spread with time. Quantitation of infectious virus produced after low MOI infection reflected this, in that while the E90ΔRL1 mutant does replicate, the amount of infectious progeny produced at 24 hrs PI is significantly less than that produced by wild-type BV. Since Vero cells do not produce IFN-*β* while primary mouse cells do, this suggests that deletion of the RL1 region from the BV genome renders the virus susceptible to the host IFN-*β* response. The E90ΔRL1 mutant does however effectively suppress the host IFN-*β* response when cells are infected with a high MOI, suggesting that low MOI infection with the E90ΔRL1 mutant allows adjacent uninfected cells to initiate an effective IFN-*β* response that the mutant is unable to overcome, preventing further spread of the virus. In this respect, the BV RL1 region deletion mutant appears similar to ICP34.5 mutants of HSV.

The E90ΔRL1 mutant was also tested in mice using the skin scarification model to assess what effect this region has on neurovirulence. As mentioned above, HSV mutants lacking the RL1 gene are not neurovirulent in mice and fail to spread within the nervous system. In contrast, the neurovirulence of the E90ΔRL1 mutant was actually slightly increased relative to that of the parental wild-type virus, the mutant having an LD_50_ of 10^3.7^ PFU compared to 10^4.4^ PFU for the parental virus. Even so, the time to death/euthanasia of infected mice was the same for both viruses (5.5–6 days PI). Thus, the RL1 region of BV does not appear to encode all the same functions ascribed to the *γ*34.5 protein of HSV and is not a major determinant of BV neurovirulence.

## 7. What Else Could Underlie the Extreme Neurovirulence of BV in Non-Macaque Hosts?

One possibility that has been raised to explain the restriction of zoonotic BV cases to North America is that some BV isolates circulating in these captive macaques are potentially recombinant viruses, the recombinant viruses having arisen due to past practices of cocaging monkeys of different species during importation. Sequencing of multiple BV isolates from different US rhesus breeding colonies failed to detect any such recombinants [77]. However, none of the isolates examined were from zoonotic BV cases. Comparative genome sequencing of BV isolates from zoonotic cases and from monkeys could determine if there are specific characteristics (including recombinants) that are associated with zoonotic isolates but not present in all monkey isolates. In addition, sequencing of BV isolated from free-ranging (noncaptive) monkeys would serve to identify the presence of recombinant viruses in North American captive macaques.

In assessing the host-specific nature of BV neurovirulence, differences that occur in how the virus behaves in the natural versus nonnatural host obviously need to be examined. There are really two critical points where differences in host-virus interactions in humans and macaques would most probably affect neurovirulence/clinical disease. The first is the ability of BV to invade the nervous system. In the normal course of pathogenesis, viral replication in epithelial tissue at the site of inoculation is needed to produce sufficient levels of infectious progeny virus to allow invasion of sensory neurons innervating the site (this is not necessary if sufficiently high levels of infectious virus are transmitted to allow direct infection of sensory neurons) [148–151]. Second, once within a sensory neuron, the linked processes of viral replication, establishment of latency, and reactivation from latency could affect the spread of BV within the nervous system.

*Replication at the Site of Infection. *The occurrence of herpetic lesions at injury sites in zoonotic BV patients suggests that BV can replicate effectively in human epithelial tissue. However, the rarity of zoonotic BV infections relative to the number of annual exposure incidents that occur and the apparent lack or rarity of asymptomatic zoonotic infections could indicate that zoonotic transmission of BV does not always lead to an active or clinically apparent infection in many or even most cases. This could be due to any number of things including (1) monkeys not shedding virus at the time of contact; (2) too low levels of virus transmitted to overcome preexisting (cross-reactive) immunity to HSV; or (3) innate host resistance resulting in inefficient or abortive replication of BV in human epithelial cells relative to its ability to replicate in macaque cells. While it has been shown that HSV replication in rhesus cells can be very inefficient or abortive [152–154], such has not been shown for BV replication in human cells. A failure of BV to replicate well in humans at the original site of infection resulting in insufficient levels of virus to invade sensory neurons could result in the majority of exposure incidents not leading to active zoonotic BV infections, such cases only resulting when high enough levels of infectious virus are transmitted to allow infection of sensory neurons without need for further amplification of virus by local replication.

Recent studies have begun to investigate the comparative replication of BV in macaque versus human cells. It has been shown that while the HSV gD glycoprotein binds host cell proteins HVEM, nectin 1 and nectin 2 to facilitate viral entry, the BV gD glycoprotein only binds nectin 1 and nectin 2 in both human and macaque cells [121, 122, 155]. While a single amino acid mutation was identified in the external domain of gD that affected the stability of binding to nectin 1, comparison of gD sequences from 19 clinical BV strains isolated from humans and macaques found that this mutation did not correlate with zoonosis [117], indicating that differing stability of gD binding to nectin 1 is not related to the ability of BV to infect human versus macaque cells. Consistent with this, the gD glycoprotein has been shown to be altogether dispensable for BV entry into both macaque and human cells [155].

The HSV IE protein ICP47 (US12) mediates downregulation of MHC expression on the surface of infected cells, thus likely evading activation of host antiviral T cells and altering susceptibility of infected cells to killing by natural killer (NK) cells [156]. The BV ICP47 protein lacks the TAP-binding domain of the HSV ICP47 and thus BV fails to downregulate MHC expression in both human and macaque cells, suggesting that the differences in BV and HSV evasion of activation of antiviral T cells and susceptibility to NK cells are not responsible for any differential pathogenicity of BV in humans versus macaques [157]. Similarly, PI3K-dependent Akt phosphorylation (which promotes survival of the infected cell and inhibits apoptosis) is not markedly different in macaque and human fibroblasts [158]. Thus, there is no evidence to date to suggest that BV replicates much differently in human versus macaque epithelial cells.

*Replication, Latency, and Reactivation in Neurons. *Virtually nothing is known regarding details of the establishment of BV latency or reactivation from latency. However, based on what is known about HSV, some predictions can be made about what might occur differently in these aspects of BV in its natural versus nonnatural host. When HSV infects a neuron, the viral envelope fuses with the cell plasma membrane, releasing the nucleocapsid and tegument proteins into the cytoplasm. The nucleocapsid (or at least the viral DNA) is transported to the nucleus where the viral DNA is immediately coated with histones and cellular repressor proteins to prevent viral IE gene expression and entry into the lytic replication cycle, resulting in latency [159, 160]. To counteract this, the viral tegument protein VP16 (UL48) recruits several host cell proteins to form complexes at IE gene promotors that facilitate expression of the viral IE genes and progression into the lytic replication cycle [159]. At the same time, the ICP0 (RL2) IE protein, which is also present in the tegument, degrades host cell proteins involved in repressing viral gene expression via its E3 ubiquitin ligase activity. ICP0 also competitively binds to certain components of repressor complexes, thus destabilizing the repressor complexes and tilting the balance against entry into the lytic replication cycle and favoring latency [123, 161]. This fine balance between lytic replication in neurons and establishment of latency represents an exquisite degree of coadaptation between HSV and its human host, allowing both to coexist. It is likely that BV follows this same paradigm in macaques, becoming latent in sensory neurons with the host adaptive immune response clearing the initial infection in epithelial tissue. Anything that alters this fine balance between lytic replication and repression of the BV genome in human neurons could well result in a very different outcome of infection compared to that seen in macaques.

As mentioned previously, there is evidence supporting the ability of BV to establish latent infections in humans and to reactivate at later times just as occurs in macaques. Even so, slight alterations in the most basic molecular aspects of these processes could have a radical effect on the ultimate outcome of BV infections in humans. It may be that in human neurons BV usually overcomes the innate preemptive response of the neuron to effectively repress viral IE gene expression and establish latency. This would result in lytic replication of BV in human neurons with production of progeny virus and subsequent spread of the virus to other neurons and/or accessory cells. Given the apparently central role played by the multifunctional ICP0 protein in the establishment of latency [123, 161], variant interaction of the BV ICP0 protein with macaque versus human cell factors could well be a critical factor.

When reactivated from latency, progeny virions (or virion components) are transported anterograde down the axon where the virus exits the neuron and undergoes lytic replication in epithelial tissue [148, 162]. While details of how reactivation from latency occurs are not known, reactivation undoubtedly once again involves altered interactions between the latent viral genome and host cell factors affecting IE gene transcription. Since HSV latency associated transcripts (LAT) and both small RNAs and miRNAs appear to be primarily involved in maintaining latency [138, 160], differential expression and/or function of homologous BV RNAs in macaque versus human neurons could alter the stability of the latent state in the two species. While some miRNAs and small RNAs have been identified and mapped in BV [103, 104], nothing is known about BV LATs or functions of these RNAs.

Since reactivation from latency must involve replication of the virus in the neuron, it has been hypothesized that replication and production of progeny virus in neurons is much less efficient than in epithelial cells, thereby sparing widespread destruction of host neurons [163]. This could well be the case in macaques, but not when BV reactivates in human neurons. Instead, BV may replicate much more efficiently in human neurons compared to macaque neurons, resulting in production of more virus within the nervous system and more efficient spread of BV within the human nervous system as seen in fatal zoonotic infections.

A similar possibility is that while BV may become latent in humans, it is reactivated much more readily in humans than in macaques (days as opposed to months or years). This would be consistent with the variable time (days to several weeks) between exposure incidents and the first appearance of clinical symptoms in zoonotic BV patients [5, 46, 51]. In the natural host, it is likely that only small amounts of infectious virus are produced in neurons following reactivation from latency and progeny virions are transported anterograde in the axon down to the original epithelial site of infection where further replication occurs until cleared by an adaptive immune response. If reactivation of BV from latency is much more efficient in human neurons, it is conceivable that BV could eventually overload the host/virus balance with successive waves of newly replicated virus infecting many more neurons than occurs in the natural macaque host.

Another possibility is that in humans BV is more readily transported down dendrites than in macaques, resulting in more efficient spread of the virus within the human nervous system. Interestingly, in pseudorabies virus (PRV; a porcine alphaherpesvirus), there does appear to be some virus-specificity in attachment of virions to dynein motor proteins (for axonal transport) versus kinesin motor proteins (for axonal and/or dendritic transport) [164]. Binding to kinesin motors involves the gE (US8), gI (US7), and US9 proteins of PRV, while tegument proteins UL36, UL37, and US3 impart affinity for dynein motors. Thus, even slightly altered specificity of any of the homologous BV proteins for macaque versus human dynein or kinesin motor proteins could affect the neurovirulence of BV in humans by altering the spread of infection from that which occurs in the natural macaque host. The failure to establish latency in human neurons, altered stability of latency, and altered spread of the virus within the nervous system are all consistent with the very serious CNS infection versus no infection picture of zoonotic BV infection that seems to occur in humans.

## 8. Conclusions

BV is ubiquitous in populations of captive and free-ranging macaques and despite many exposure incidents every year; zoonotic infections are extremely rare and have only been documented in North America. Notwithstanding, BV is notorious for its extreme neurovirulence in the handful of humans who have been infected. Biological aspects of BV infection in its natural macaque host are very similar to that of HSV in humans, including primary replication in epithelial tissue, invasion of sensory neurons, establishment of latency in sensory ganglia, and periodic reactivation from latency in response to stress allowing transmission of the virus to a new host. Phylogenetic analysis of the primate alphaherpesviruses suggests that these viruses have likely coevolved with their hosts, not surprising given the exquisite details that must be involved in virus-host interactions to maintain the virus within the nervous system for the lifespan of its host without serious adverse consequences while still allowing transmission and perpetuation of the virus within the host species. Given that nonhuman primates are our closest phylogenetic relatives, it is not surprising that BV should be very much like HSV with regard to the virus-host interactions and mechanisms involved in maintaining the virus-host relationship in a balanced state. The orthologous nature of the BV and HSV genomes and similarity of encoded proteins support this. Based on comparative sequence analyses, it also appears that BV encodes LATs, miRNAs, and small RNAs that would be involved in the intricate regulation of viral latency in neurons as in HSV.

Due to the hazardous and restrictive nature of performing research with infectious BV, very little experimental or molecular research has been done on this intriguing virus. To date the only genetic characteristic that differs dramatically between BV and HSV is the lack of homologs of the RL1 (*γ*34.5) and ORF P genes in BV. Despite lacking these genes, this region of the simian virus genome remains nearly the same size as in HSV. Although both the RL1 and ORF P genes are important determinants of HSV neurovirulence in mice, deletion of this region of the BV genome has little effect on its neurovirulence in mice.

BV has attained its reputation as having extreme neurovirulence due to the high mortality associated with the approximately 60 zoonotic cases that have occurred since its identification 85 years ago. However, for comparison HSV causes ~500 cases of encephalitis each year in the US, and without treatment is ~70% fatal with only ~5% of patients fully recovering [18], characteristics that are very similar to that of zoonotic BV. The neurovirulence of BV is only apparent when it infects non-macaques. While there are some differences between HSV and BV, to date no differences in the replication of BV* in vitro* in human versus macaque cells have been identified that might account for the divergent neurovirulence of BV in these two species. Given the delicate balance that exists in neurons between repression of gene expression to establish latency and lytic replication, even slight differences in any of the multitude of host-virus interactions affecting this balance could ultimately alter the pathogenesis of BV in a nonnatural host species, resulting in very different outcomes of infection in macaques versus humans. Considerably more research into molecular aspects of host-virus interactions in macaque versus human cells, both epithelial and neural, needs to be pursued to better understand the extreme neurovirulence exhibited by BV in humans.
